# Diethyl 2-amino-5-[(*E*)-(furan-2-yl­methyl­idene)amino]­thio­phene-3,4-di­carboxyl­ate

**DOI:** 10.1107/S1600536810043746

**Published:** 2010-10-31

**Authors:** Stéphane Dufresne, W. G. Skene

**Affiliations:** a Department of Chemistry, University of Montreal, CP 6128, succ. Centre-ville, Montréal, Québec, Canada H3C 3J7

## Abstract

In the crystal structure of the title compound, C_15_H_16_N_2_O_5_S, the azomethine adopts the *E* configuration. The two heterocyclic rings adopt an anti­periplanar orientation. The mean planes of the thio­phene and furan rings are twisted by 2.51 (4)°. The crystal structure exhibits inter­molecular N—H⋯O hydrogen bonding. π–π stacking is also observed, the centroid-to-centroid distance being 3.770 (4) Å.

## Related literature

For general background, see: Dufresne & Skene (2008 ▶). For a related crystal structure, see: Skene *et al.* (2006 ▶)
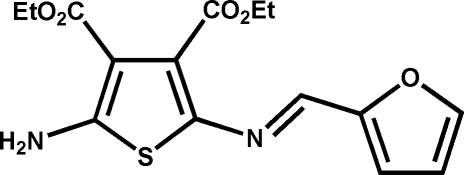


## Experimental

### 

#### Crystal data

C_15_H_16_N_2_O_5_S
*M* *_r_* = 336.36
Monoclinic, 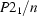

*a* = 9.3452 (19) Å
*b* = 14.635 (3) Å
*c* = 11.343 (2) Å
β = 99.73 (3)°
*V* = 1529.0 (5) Å^3^
*Z* = 4
Cu *K*α radiation
μ = 2.14 mm^−1^
*T* = 123 K
0.14 × 0.10 × 0.04 mm

#### Data collection

Bruker SMART 6000 diffractometer
Absorption correction: multi-scan (*SADABS*; Sheldrick, 1996 ▶) *T*_min_ = 0.728, *T*_max_ = 0.920
6320 measured reflections
3005 independent reflections
2475 reflections with *I* > 2σ(*I*)
*R*_int_ = 0.036

#### Refinement

*R*[*F* ^2^ > 2σ(*F* ^2^)] = 0.037
*wR*(*F* ^2^) = 0.102
*S* = 1.03
3005 reflections
210 parameters
H-atom parameters constrained
Δρ_max_ = 0.29 e Å^−3^
Δρ_min_ = −0.34 e Å^−3^

### 

Data collection: *SMART* (Bruker, 2003 ▶); cell refinement: *SAINT* (Bruker, 2004 ▶); data reduction: *SAINT*; program(s) used to solve structure: *SHELXS97* (Sheldrick, 2008 ▶); program(s) used to refine structure: *SHELXL97* (Sheldrick, 2008 ▶); molecular graphics: *SHELXTL* (Sheldrick, 2008 ▶) and *ORTEP-3* (Farrugia, 1997 ▶); software used to prepare material for publication: *UdMX* (Marris, 2004 ▶).

## Supplementary Material

Crystal structure: contains datablocks I, global. DOI: 10.1107/S1600536810043746/wn2415sup1.cif

Structure factors: contains datablocks I. DOI: 10.1107/S1600536810043746/wn2415Isup2.hkl

Additional supplementary materials:  crystallographic information; 3D view; checkCIF report

## Figures and Tables

**Table 1:** Hydrogen-bond geometry (Å, °)

*D*—H⋯*A*	*D*—H	H⋯*A*	*D*⋯*A*	*D*—H⋯*A*
N1—H1*A*⋯O2^i^	0.88	2.20	2.889 (2)	135
N1—H1*B*⋯O4^ii^	0.88	2.50	3.059 (3)	122

Symmetry codes: (i) 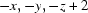; (ii) 
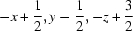
.

**Fig. 1.**
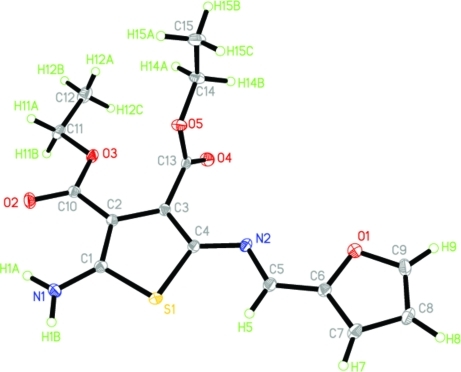
ORTEP-3 (Farrugia, 1997) representation of the molecular structure, with the numbering scheme adopted. Displacement ellipsoids are drawn at the 30% probability level. H atoms are shown as spheres of arbitrary radius.

**Fig. 2.**
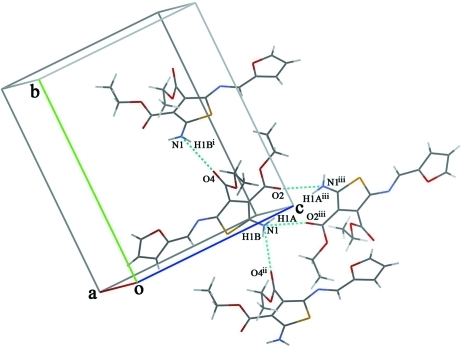
Supramolecular structure showing the intermolecular hydrogen bonding (dashed lines). [Symmetry codes: (i) 1/2 - x, 1/2 + y, 1.5 - z; (ii) 1/2 - x, -1/2 + y, 1.5 - z; (iii) -x, -y, 2 - z.]

**Fig. 3.**
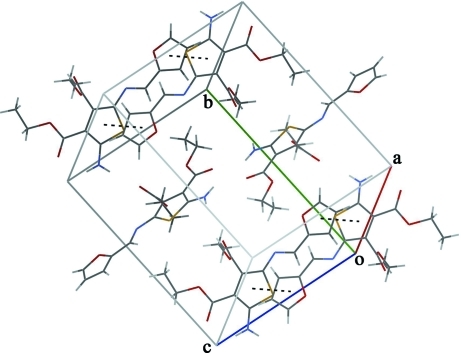
The three-dimensional network demonstrating the π-stacking (dashed lines) in the crystal structure.

## supplementary crystallographic information

### Comment

During the course of our ongoing conjugated azomethine research, we prepared the title compound. The X-ray crystallographic analysis not only confirmed the structure (Fig. 1), but that the energetically stable E isomer was formed. Neither solvent nor counter-ions were found in the structure.

The heterocyclic rings were found not to be coplanar; the angle between the heterocyclic mean planes is 2.51 (4)°. This angle is less than that in a previously reported azomethine thiophene system, whose angle is 7.25 (11)° (Skene *et al.*, 2006).

A major point of interest is the azomethine bond. The bond lengths for N2—C4, N2—C5 and C5—C6 are 1.382 (2), 1.289 (2) and 1.420 (2) Å, respectively. These are similar to the related azomethine thiophene compound (Skene *et al.*, 2006), whose homologous lengths are 1.381 (3), 1.283 (3) and 1.426 (3) Å.

Fig. 2 shows that two different hydrogen bonds occur in the crystal structure, viz. N1—H1A···O2^iii^ and N1—H1B···O4^ii^. The D—H···A angles are 135° and 122° and distances of 2.880 (3) Å and 3.059 (3) Å were measured between the nitrogen and oxygens (Table 1). Dimerization of two molecules occurs *via* H-bonding between N1—H1A···O2^iii^. Additionally, π-stacking takes place between two different molecules, at [*x*, *y*, *z*] and [1 - *x*, -*y*, 1 - *z*]. Fig. 3 shows the interactions, with the distance between the planes being 3.440 (4) Å. The centroid···centroid distance between the two rings is 3.770 (4) Å.

### Experimental

2-Furaldehyde (37 mg, 0.39 mmol) and 2,5-diamino-thiophene-3,4-dicarboxylic acid diethyl ester (100 mg, 0.39 mmol) were mixed in anhydrous 2-propanol with a catalytic amount of TFA and refluxed for 12 h. The reaction was then purified by flash chromatography to afford the title compound as a brownish yellow solid (110 mg, 85%). Single crystals were obtained by slow evaporation of an acetone solution.

### Refinement

Carbon-bound H atoms were placed in calculated positions (Cmethyl—H = 0.98 Å, Cmethylene—H = 0.99 Å and Csp^2^—H = 0.95 Å) and included in the refinement in the riding-model approximation, with *U*_iso_(H) = k*U*_eq_(C), where k = 1.5 for Cmethyl and 1.2 for Cmethylene and Csp^2^. The H atoms of the amino group were placed in calculated positions (N—H = 0.88 Å) and included in the refinement in the riding-model approximation, with *U*_iso_(H) = 1.2*U*_eq_(N).

### Crystal data

**Table:**

C_15_H_16_N_2_O_5_S	*F*(000) = 704
*M**_r_* = 336.36	*D*_x_ = 1.461 Mg m^−^^3^
Monoclinic, *P*2_1_/*n*	Melting point: 425(2) K
Hall symbol: -P 2yn	Cu *K*α radiation, λ = 1.54178 Å
*a* = 9.3452 (19) Å	Cell parameters from 3360 reflections
*b* = 14.635 (3) Å	θ = 5.0–38.8°
*c* = 11.343 (2) Å	µ = 2.14 mm^−^^1^
β = 99.73 (3)°	*T* = 123 K
*V* = 1529.0 (5) Å^3^	Block, yellow
*Z* = 4	0.14 × 0.10 × 0.04 mm

### Data collection

**Table:**

Bruker SMART 6000 diffractometer	3005 independent reflections
Radiation source: rotating anode	2475 reflections with *I* > 2σ(*I*)
Montel 200 optics	*R*_int_ = 0.036
Detector resolution: 5.5 pixels mm^-1^	θ_max_ = 72.3°, θ_min_ = 5.0°
ω scans	*h* = −11→11
Absorption correction: multi-scan (*SADABS*; Sheldrick, 1996)	*k* = −17→18
*T*_min_ = 0.728, *T*_max_ = 0.920	*l* = −13→13
6320 measured reflections	

### Refinement

**Table:**

Refinement on *F*^2^	Primary atom site location: structure-invariant direct methods
Least-squares matrix: full	Secondary atom site location: difference Fourier map
*R*[*F*^2^ > 2σ(*F*^2^)] = 0.037	Hydrogen site location: inferred from neighbouring sites
*wR*(*F*^2^) = 0.102	H-atom parameters constrained
*S* = 1.03	*w* = 1/[σ^2^(*F*_o_^2^) + (0.0643*P*)^2^] where *P* = (*F*_o_^2^ + 2*F*_c_^2^)/3
3005 reflections	(Δ/σ)_max_ = 0.001
210 parameters	Δρ_max_ = 0.29 e Å^−^^3^
0 restraints	Δρ_min_ = −0.34 e Å^−^^3^

### Special details

**Table:**

Geometry. All e.s.d.'s (except the e.s.d. in the dihedral angle between two l.s. planes) are estimated using the full covariance matrix. The cell e.s.d.'s are taken into account individually in the estimation of e.s.d.'s in distances, angles and torsion angles; correlations between e.s.d.'s in cell parameters are only used when they are defined by crystal symmetry. An approximate (isotropic) treatment of cell e.s.d.'s is used for estimating e.s.d.'s involving l.s. planes.
Refinement. Refinement of *F*^2^ against ALL reflections. The weighted *R*-factor *wR* and goodness of fit *S* are based on *F*^2^, conventional *R*-factors *R* are based on *F*, with *F* set to zero for negative *F*^2^. The threshold expression of *F*^2^ > σ(*F*^2^) is used only for calculating *R*-factors(gt) *etc*. and is not relevant to the choice of reflections for refinement. *R*-factors based on *F*^2^ are statistically about twice as large as those based on *F*, and *R*-factors based on ALL data will be even larger.

### Fractional atomic coordinates and isotropic or equivalent isotropic displacement parameters (Å^2^)

**Table:**

	*x*	*y*	*z*	*U*_iso_*/*U*_eq_	
S1	0.15876 (5)	0.01058 (3)	0.62132 (4)	0.02153 (13)	
O3	0.26457 (13)	0.20322 (8)	0.97617 (10)	0.0216 (3)	
O5	0.53448 (13)	0.16250 (8)	0.86520 (11)	0.0229 (3)	
O1	0.53601 (14)	0.14839 (10)	0.36188 (11)	0.0299 (3)	
O4	0.42258 (13)	0.28091 (8)	0.76310 (11)	0.0261 (3)	
O2	0.11370 (14)	0.08821 (9)	1.00209 (11)	0.0280 (3)	
N2	0.37833 (15)	0.11513 (10)	0.55344 (12)	0.0201 (3)	
N1	0.01952 (16)	−0.02136 (10)	0.80267 (14)	0.0247 (3)	
H1A	−0.0029	−0.0108	0.8737	0.030*	
H1B	−0.0274	−0.0637	0.7563	0.030*	
C13	0.42543 (18)	0.20165 (12)	0.79259 (14)	0.0180 (3)	
C2	0.21155 (17)	0.09610 (11)	0.82328 (15)	0.0177 (3)	
C4	0.29499 (18)	0.09523 (12)	0.63985 (15)	0.0194 (4)	
C10	0.19189 (18)	0.12672 (12)	0.94126 (15)	0.0192 (4)	
C3	0.30893 (17)	0.13360 (12)	0.75044 (14)	0.0175 (3)	
C14	0.65332 (19)	0.22184 (13)	0.91629 (17)	0.0274 (4)	
H14A	0.6231	0.2619	0.9780	0.033*	
H14B	0.6830	0.2608	0.8533	0.033*	
C1	0.12570 (18)	0.02730 (12)	0.76478 (15)	0.0191 (4)	
C6	0.42538 (19)	0.08612 (12)	0.35493 (16)	0.0218 (4)	
C5	0.35120 (19)	0.07232 (12)	0.45285 (15)	0.0225 (4)	
H5	0.2757	0.0281	0.4435	0.027*	
C11	0.2356 (2)	0.24369 (13)	1.08714 (15)	0.0259 (4)	
H11A	0.2526	0.1984	1.1528	0.031*	
H11B	0.1335	0.2646	1.0776	0.031*	
C8	0.5077 (2)	0.08255 (14)	0.18213 (16)	0.0284 (4)	
H8	0.5204	0.0675	0.1031	0.034*	
C7	0.4049 (2)	0.04441 (13)	0.24649 (16)	0.0272 (4)	
H7	0.3351	−0.0014	0.2191	0.033*	
C9	0.5836 (2)	0.14410 (15)	0.25473 (17)	0.0316 (5)	
H9	0.6604	0.1800	0.2343	0.038*	
C15	0.7766 (2)	0.16205 (14)	0.97085 (18)	0.0320 (4)	
H15A	0.7455	0.1231	1.0321	0.048*	
H15B	0.8583	0.2002	1.0075	0.048*	
H15C	0.8068	0.1237	0.9087	0.048*	
C12	0.3367 (2)	0.32281 (13)	1.11445 (17)	0.0351 (5)	
H12A	0.4372	0.3007	1.1284	0.053*	
H12B	0.3167	0.3543	1.1862	0.053*	
H12C	0.3227	0.3653	1.0467	0.053*	

### Atomic displacement parameters (Å^2^)

**Table:**

	*U*^11^	*U*^22^	*U*^33^	*U*^12^	*U*^13^	*U*^23^
S1	0.0226 (2)	0.0245 (2)	0.0170 (2)	−0.00387 (17)	0.00208 (16)	−0.00373 (16)
O3	0.0288 (7)	0.0215 (6)	0.0158 (6)	−0.0027 (5)	0.0072 (5)	−0.0041 (5)
O5	0.0206 (6)	0.0213 (6)	0.0245 (7)	−0.0023 (5)	−0.0025 (5)	−0.0003 (5)
O1	0.0275 (7)	0.0373 (8)	0.0254 (7)	−0.0063 (6)	0.0062 (6)	−0.0053 (6)
O4	0.0286 (7)	0.0206 (7)	0.0278 (7)	−0.0032 (5)	0.0013 (5)	0.0036 (5)
O2	0.0310 (7)	0.0308 (7)	0.0253 (7)	−0.0070 (6)	0.0134 (6)	−0.0019 (6)
N2	0.0221 (7)	0.0234 (8)	0.0148 (7)	0.0023 (6)	0.0036 (6)	0.0009 (6)
N1	0.0242 (8)	0.0269 (8)	0.0239 (8)	−0.0083 (6)	0.0069 (6)	−0.0038 (6)
C13	0.0205 (8)	0.0216 (9)	0.0131 (8)	0.0010 (6)	0.0060 (6)	−0.0011 (6)
C2	0.0171 (8)	0.0183 (8)	0.0175 (8)	0.0003 (6)	0.0028 (6)	0.0004 (6)
C4	0.0191 (8)	0.0222 (9)	0.0170 (8)	0.0003 (7)	0.0032 (7)	0.0008 (7)
C10	0.0178 (8)	0.0213 (9)	0.0184 (8)	0.0015 (6)	0.0025 (7)	0.0006 (7)
C3	0.0174 (8)	0.0184 (8)	0.0168 (8)	0.0013 (6)	0.0032 (6)	0.0017 (6)
C14	0.0222 (9)	0.0273 (10)	0.0309 (10)	−0.0057 (7)	−0.0011 (8)	−0.0059 (8)
C1	0.0181 (8)	0.0200 (8)	0.0185 (8)	0.0023 (6)	0.0013 (7)	0.0006 (7)
C6	0.0235 (8)	0.0226 (9)	0.0188 (8)	0.0000 (7)	0.0022 (7)	0.0008 (7)
C5	0.0267 (9)	0.0222 (9)	0.0188 (9)	−0.0013 (7)	0.0041 (7)	0.0005 (7)
C11	0.0380 (10)	0.0256 (9)	0.0154 (8)	0.0023 (8)	0.0080 (8)	−0.0038 (7)
C8	0.0314 (10)	0.0375 (11)	0.0174 (9)	0.0075 (8)	0.0076 (8)	0.0002 (8)
C7	0.0365 (10)	0.0259 (10)	0.0198 (9)	−0.0008 (8)	0.0061 (8)	−0.0038 (7)
C9	0.0266 (10)	0.0429 (12)	0.0278 (10)	−0.0014 (8)	0.0115 (8)	0.0052 (9)
C15	0.0236 (9)	0.0391 (11)	0.0303 (10)	0.0003 (8)	−0.0043 (8)	−0.0037 (9)
C12	0.0578 (14)	0.0241 (10)	0.0235 (10)	−0.0043 (10)	0.0078 (9)	−0.0049 (8)

### Geometric parameters (Å, °)

**Table:**

S1—C1	1.7242 (18)	C14—C15	1.495 (3)
S1—C4	1.7633 (18)	C14—H14A	0.99
O3—C10	1.334 (2)	C14—H14B	0.99
O3—C11	1.4573 (19)	C6—C7	1.357 (2)
O5—C13	1.3272 (19)	C6—C5	1.420 (2)
O5—C14	1.451 (2)	C5—H5	0.95
O1—C9	1.364 (2)	C11—C12	1.494 (3)
O1—C6	1.370 (2)	C11—H11A	0.99
O4—C13	1.206 (2)	C11—H11B	0.99
O2—C10	1.224 (2)	C8—C9	1.340 (3)
N2—C5	1.289 (2)	C8—C7	1.416 (3)
N2—C4	1.382 (2)	C8—H8	0.95
N1—C1	1.349 (2)	C7—H7	0.95
N1—H1A	0.88	C9—H9	0.95
N1—H1B	0.88	C15—H15A	0.98
C13—C3	1.493 (2)	C15—H15B	0.98
C2—C1	1.385 (2)	C15—H15C	0.98
C2—C3	1.437 (2)	C12—H12A	0.98
C2—C10	1.452 (2)	C12—H12B	0.98
C4—C3	1.360 (2)	C12—H12C	0.98
			
C1—S1—C4	91.65 (8)	C7—C6—C5	129.21 (18)
C10—O3—C11	115.96 (13)	O1—C6—C5	120.86 (16)
C13—O5—C14	116.42 (14)	N2—C5—C6	125.07 (17)
C9—O1—C6	105.92 (15)	N2—C5—H5	117.5
C5—N2—C4	118.38 (15)	C6—C5—H5	117.5
C1—N1—H1A	120	O3—C11—C12	106.90 (15)
C1—N1—H1B	120	O3—C11—H11A	110.3
H1A—N1—H1B	120	C12—C11—H11A	110.3
O4—C13—O5	124.46 (16)	O3—C11—H11B	110.3
O4—C13—C3	124.88 (16)	C12—C11—H11B	110.3
O5—C13—C3	110.65 (14)	H11A—C11—H11B	108.6
C1—C2—C3	111.96 (15)	C9—C8—C7	106.42 (17)
C1—C2—C10	120.84 (15)	C9—C8—H8	126.8
C3—C2—C10	127.08 (15)	C7—C8—H8	126.8
C3—C4—N2	126.11 (16)	C6—C7—C8	106.60 (17)
C3—C4—S1	110.77 (13)	C6—C7—H7	126.7
N2—C4—S1	123.06 (13)	C8—C7—H7	126.7
O2—C10—O3	122.79 (16)	C8—C9—O1	111.14 (17)
O2—C10—C2	123.91 (16)	C8—C9—H9	124.4
O3—C10—C2	113.27 (14)	O1—C9—H9	124.4
C4—C3—C2	113.65 (15)	C14—C15—H15A	109.5
C4—C3—C13	121.43 (15)	C14—C15—H15B	109.5
C2—C3—C13	124.67 (15)	H15A—C15—H15B	109.5
O5—C14—C15	107.41 (15)	C14—C15—H15C	109.5
O5—C14—H14A	110.2	H15A—C15—H15C	109.5
C15—C14—H14A	110.2	H15B—C15—H15C	109.5
O5—C14—H14B	110.2	C11—C12—H12A	109.5
C15—C14—H14B	110.2	C11—C12—H12B	109.5
H14A—C14—H14B	108.5	H12A—C12—H12B	109.5
N1—C1—C2	128.96 (16)	C11—C12—H12C	109.5
N1—C1—S1	118.97 (13)	H12A—C12—H12C	109.5
C2—C1—S1	111.94 (13)	H12B—C12—H12C	109.5
C7—C6—O1	109.92 (16)		
			
C14—O5—C13—O4	3.5 (2)	O5—C13—C3—C4	−101.12 (18)
C14—O5—C13—C3	−177.89 (14)	O4—C13—C3—C2	−108.6 (2)
C5—N2—C4—C3	179.86 (17)	O5—C13—C3—C2	72.8 (2)
C5—N2—C4—S1	2.9 (2)	C13—O5—C14—C15	−167.31 (15)
C1—S1—C4—C3	−1.07 (14)	C3—C2—C1—N1	−177.66 (17)
C1—S1—C4—N2	176.35 (15)	C10—C2—C1—N1	−1.3 (3)
C11—O3—C10—O2	−5.4 (2)	C3—C2—C1—S1	−1.82 (18)
C11—O3—C10—C2	172.78 (14)	C10—C2—C1—S1	174.49 (12)
C1—C2—C10—O2	9.1 (3)	C4—S1—C1—N1	177.96 (14)
C3—C2—C10—O2	−175.17 (17)	C4—S1—C1—C2	1.66 (13)
C1—C2—C10—O3	−169.01 (15)	C9—O1—C6—C7	0.0 (2)
C3—C2—C10—O3	6.7 (2)	C9—O1—C6—C5	179.52 (17)
N2—C4—C3—C2	−177.09 (15)	C4—N2—C5—C6	178.96 (16)
S1—C4—C3—C2	0.22 (19)	C7—C6—C5—N2	179.79 (19)
N2—C4—C3—C13	−2.6 (3)	O1—C6—C5—N2	0.3 (3)
S1—C4—C3—C13	174.76 (12)	C10—O3—C11—C12	175.14 (15)
C1—C2—C3—C4	1.0 (2)	O1—C6—C7—C8	−0.1 (2)
C10—C2—C3—C4	−175.00 (16)	C5—C6—C7—C8	−179.58 (18)
C1—C2—C3—C13	−173.30 (15)	C9—C8—C7—C6	0.2 (2)
C10—C2—C3—C13	10.7 (3)	C7—C8—C9—O1	−0.2 (2)
O4—C13—C3—C4	77.5 (2)	C6—O1—C9—C8	0.1 (2)

### Hydrogen-bond geometry (Å, °)

**Table:**

*D*—H···*A*	*D*—H	H···*A*	*D*···*A*	*D*—H···*A*
N1—H1A···O2^i^	0.88	2.20	2.889 (2)	135
N1—H1B···O4^ii^	0.88	2.50	3.059 (3)	122

Symmetry codes: (i) −*x*, −*y*, −*z*+2; (ii) −*x*+1/2, *y*−1/2, −*z*+3/2.

## References

[bb1] Bruker (2003). *SMART.* Bruker AXS Inc., Madison, Wisconsin, USA.

[bb2] Bruker (2004). *SAINT.* Bruker AXS Inc., Madison, Wisconsin, USA.

[bb3] Dufresne, S. & Skene, W. G. (2008). *J. Org. Chem.* **73**, 3859–3866. 10.1021/jo8002503 18410143

[bb4] Farrugia, L. J. (1997). *J. Appl. Cryst.* **30**, 565.

[bb5] Marris, T. (2004). *UdMX* Université de Montréal, Canada.

[bb6] Sheldrick, G. M. (1996). *SADABS* University of Göttingen, Germany.

[bb7] Sheldrick, G. M. (2008). *Acta Cryst.* A**64**, 112–122. 10.1107/S0108767307043930 18156677

[bb8] Skene, W. G., Dufresne, S., Trefz, T. & Simard, M. (2006). *Acta Cryst.* E**62**, o2382–o2384.

